# Impact of Technological Processes on the Formation of Furosine, Acrylamide and Furan in Traditional Venezuelan Cocoa

**DOI:** 10.3390/foods13060829

**Published:** 2024-03-08

**Authors:** Thayra Rocio Moreno-Trujillo, Elevina Perez, Vito Verardo, Belén García-Villanova, Eduardo Jesús Guerra-Hernández

**Affiliations:** 1 Department of Nutrition and Food Science, School of Pharmacy, University of Granada, Cartuja Campus, 18071 Granada, Spain; thayrarocio@gmail.com (T.R.M.-T.); belenv@ugr.es (B.G.-V.); ejguerra@ugr.es (E.J.G.-H.); 2 Instituto de Ciencia y Tecnología de Alimentos (ICTA), Facultad de Ciencias, Universidad Central de Venezuela, Apartado Postal 47097 Los Chaguaramos, Caracas 1041-A, Venezuela; elevina07@gmail.com; 3 Institute of Nutrition and Food Technology “José Mataix”, Biomedical Research Centre, University of Granada, Avda del Conocimiento s/n., 18100 Granada, Spain

**Keywords:** *Theobroma cacao*, roasting, alkalisation, furosine, acrylamide, furan

## Abstract

The present study was conducted to determine and analyse the content of furosine, acrylamide and furan in fermented cocoa beans from the Chuao (“criollo variety”) and Barlovento (“trinitario variety”) regions of Venezuela, after roasting (in the shell at 110–180 °C for 15–60 min) and alkalisation (with sodium bicarbonate or potassium carbonate, at concentrations of 10–25 g/kg in order to evaluate the impact of these operations. The highest furosine contents (up to 249 mg/100 g of protein) were found in fermented, sun-dried samples, and were higher in the nibs than in the shells. The acrylamide content increased in line with the temperature, to 160 °C in the shells, and to 180 °C in the nibs. At temperatures of up to 140 °C, the acrylamide content was higher in the shells than in the nibs. The furan content increased in line with the temperature and in this case too, was greater in the shells. The content of both furosine and furan decreased with alkalisation, while the presence of acrylamide was irregular and determined by the roasting temperature and the alkalising agent employed. Although the furosine, acrylamide and furan contents varied between the beans from the two regions and the varieties considered (Chuao and Barlovento), these three compounds were correlated to a statistically significant degree.

## 1. Introduction

The cocoa bean is the main raw material of chocolate, and Venezuelan cocoa (*Theobroma cacao* L.) has long been prized for its unique qualities. Three main varieties of cocoa are cultivated worldwide: forastero (the most widespread), criollo and trinitario (a hybrid of the other two). The latter two types are mainly grown in Venezuela. The highest quality cocoa beans come from the regions of Chuao, Cuyagua, Barlovento and Ocumare, where the pedoclimatic conditions are optimal. These beans are processed as follows: first, they are separated manually, collected in baskets and transported to the fermentation station, where they are fermented, depending on local custom, for 3–7 days. Then they are roasted at 110 to 180 °C for 5–120 min (according to the temperature applied) [1]. Optionally, the beans may be alkalised to reduce their natural bitterness and enhance the colour, making the product darker [2].

Roasting is the most important technological operation in cocoa bean processing, and the chemical changes produced depend on the temperature applied during this process. The properties of roasted beans—their texture and colour, the concentration of volatile flavour compounds, total acidity and fat content—depend on the roasting conditions applied, especially temperature and duration [3]. During this step, the Maillard reaction (MR) takes place. This complex chemical reaction is initiated when the free amino group of the amino acids attacks the reactive carbonyl groups of reducing sugars such as glucose and fructose, giving rise to Amadori compounds, which are precursors for the formation of 3-deoxyhexulose and 2,3-enediol with dehydroreductone intermediates under acidic and basic/neutral conditions, respectively. From the latter, α-dicarbonyl compounds are formed, which then undergo Strecker degradation and heterocyclisation, generating aldehydes, ketones, pyrazines, pyrroles and pyridines, among other products [4]; hence, producing the desired chocolate flavour. The forastero variety of cocoa bean produces the “bulk” flavour, characterised by a strongly acidic, astringent, intense impact, with weaker fruity/floral tones. This variety constitutes 95% of the global harvest. The criollo variety contributes the “fine” flavour, providing fruity, floral and spicy tones [5].

Although cocoa powder is nutritive, supplying carbohydrates (58%), proteins (20%), fats (11%) and other functional constituents such as polyphenols (mainly the flavanols catechin, epicatechin and procyanidins B1 and B2) [6], its processing also generates toxic/carcinogenic components like acrylamide, furan and polycyclic aromatic hydrocarbons (PAHs) [7,8].

The International Agency for Research on Cancer (IARC) has classified acrylamide as a category 2A substance, meaning it is “probably carcinogenic” [9], while furan is classed as category 2B, “possibly carcinogenic”. Furan results from the thermal processing of food from a wide range of precursors, including amino acids, sugars, polyunsaturated fatty acids and carotenoids, and animal studies have shown it has carcinogenic and cytotoxic properties [10].

In April 2002, the Swedish National Food Administration drew attention to the risks of acrylamide generated in food processing, attracting global interest and leading to the systematic monitoring of its presence in food products [11]. Since then, concentrations ranging from 9 to 1747 µg/kg have been found in commercial samples of cocoa beans and derived products such as chocolate [12,13,14,15,16,17,18]. Roasting has been found to generate acrylamide values of up to 7800 µg/kg, when dehulled fermented cocoa beans are toasted at 150 °C for 25 min [19]. Studies have also been conducted to investigate the consequences of fermentation and drying [7,20] but to our knowledge only one study has been carried out on the relation between alkalisation and acrylamide content [9].

With respect to furan, values of up to 40 µg/kg have been measured in commercial samples of cocoa and derivatives (chocolate drinks, syrups and powders and cocoa powder) ([7,21,22,23]. Only one previous study has considered the use of furosine, a product of Amadori compound degradation, in these types of foods [24].

The aim of our study is to evaluate the impact of fermentation, roasting and alkalisation on the content of furosine, acrylamide and furan in cocoa samples obtained from the Chuao (“criollo variety”) and Barlovento (“trinitario variety”) regions of Venezuela.

## 2. Materials and Methods

### 2.1. Chemicals

Acrylamide standard (99%), potassium ferrocyanide (Carrez I) and zinc acetate (Carrez-II), heptafluorobutyric acid and methanol (HPLC grade) were purchased from Sigma-Aldrich (St. Louis, MO, USA). [^13^C_3_]-acrylamide (isotopic purity 99%) was obtained from Cambridge Isotope Labs (Andover, MA, USA). Formic acid was obtained from Merck (Darmstadt, Germany). Hydrochloric acid, glacial acetic acid, potassium chloride, sodium chloride, sodium bicarbonate, potassium carbonate, potassium sulphate, sodium sulphate, cupric sulphate, sulphuric acid, sodium hydroxide and boric acid were obtained from Panreac (Barcelona, Spain). Reversed-phase Oasis-HLB cartridges and Sep-Pack cartridges (C18) were obtained from Waters (Milford, MA, USA). Furosine was obtained from Neosystem Laboratories (Strasbourg, France). Furan (Fluka, Madrid, Spain) and furan-d4 from Isotec (Miamisburg, Ohio, USA) were obtained at a minimum purity of 98%. Demineralised water was obtained by filtering distilled water through a Milli-Q Ultrapure Water System (Millipore, Bedford, MA, USA).

### 2.2. Samples

Artisan-fermented, sun-dried cocoa beans from Chuao (criollo variety) and Barlovento (trinitario: mix of criollo and forastero variety) were analysed after the following process. Samples of approximately 200 g were oven roasted at 110 °C and 140 °C for 60 min. They were then allowed to cool and manually decorticated to obtain the nibs (decorticated beans) and shells. Additionally, 50 g samples of Barlovento beans were roasted at 160 °C for 40 and 60 min and at 180 °C for 15 and 30 min, in order to obtain over-roasted samples. All samples were then milled in a Moulinex DAE241 blender to 40 mesh.

Alkalisation. Solutions of sodium bicarbonate and potassium carbonate, at concentrations of 10, 15, 20 and 25 g/kg were used, following the recommendations of Wissgott [25]. To perform the alkalisation with each type of alkali at each concentration level, 50 g of nibs were dispersed in the corresponding solution (100 mL of solution containing 10, 15, 20 or 25 g of the alkali in 1 L water), and quantitatively transferred to a 1 L round-bottom flask and heated at 80–85 °C for 1 h. The neck of the flask was connected to a reflux condenser in order to maintain a constant volume and minimal evaporation loss. The flask was heated in a boiling water bath. Once alkalisation was attained, the resulting liquor was dried in a tray drier (Mitchell Dryers, No. 655 149, Manchester, UK) at 70 °C for 2–3 h, until constant moisture (~100 g/kg) was reached. The dried alkalised samples were then milled in a Moulinex DAE241 blender to obtain a granulometric size of approximately 40 mesh, and stored in a hermetic glass container for further analysis.

The samples were coded as follows: N = Nibs; S = Shell; C = Chuao area; B = Barlovento area; T = Temperature applied (110, 140, 160 or 180 °C); Na = Sodium bicarbonate; K = Potassium carbonate. The alkali solution concentrations used were 10, 15, 20 and 25 g/kg (1; 1.5; 2 and 2.5%) (see Table 1).

### 2.3. Furosine Assay

Furosine was determined following the method described by Resmini et al. [26] with slight modifications. A finely milled sample of 0.6 g was hydrolysed with 8 mL of 7.95 mol/L HCl at 110 °C for 23 h in a Pyrex screw-cap vial with PTFE-faced septa. High-purity N_2_ gas was bubbled through the solution for 2 min and hydrolysed at 110 °C for 24 h. A 0.5 mL portion of the filtrate was applied to a Sep-pack C18 cartridge (Waters, Milford, MA, USA) prewetted with 5 mL of methanol and 10 mL of deionised water and was then eluted with 3 mL of HCl 3M. Then, 50 microlitres of the solution were injected into a high-pressure liquid chromatography (HPLC) system, which consisted of a Waters 600 (Milford, MA, USA) and a Konic UV/VIS detector (Barcelona, Spain). Furosine was separated using a C8 furosine-dedicated column (250 × 4.6 mm) from Alltech (Nicholasville, KY, USA). The mobile phase was (A) 0.4% acetic acid/water (*v*/*v*) and (B) 0.3% potassium chloride/phase A (*w*/*v*). The binary gradient was 100% A for 20 min, 50% B for 3 min and 100% A for 9 min, and the flow rate was 1.2 mL/min. The UV/VIS detector was set at 280 nm. The chromatographic system was calibrated by the external standard method. A standard stock solution containing 0.744 mg/mL of furosine was used to prepare the working standard solution. Calibration was performed by adding increasing quantities of the furosine standard, within the expected concentration range, to a previously hydrolysed raw cocoa sample. The curve was constructed in units of area against micrograms of added furosine. The equation for the curve was Y = 8.6 × 10^6^X − 332 (range: 0.0011 to 0.151 µg), r^2^ = 0.9986, where Y is the peak area and X the µg of furosine. The results are expressed as milligrams of furosine per 100 g of protein (mg furosine/100 g of protein). The LOD and LOQ were 0.52 and 1.75 mg/100 g of protein [27].

### 2.4. Acrylamide Assay

Acrylamide was determined following the method described by Kocadağlı et al. [28] with slight modifications. A finely milled sample (0.500 g) was weighed and mixed with 5 mL of 10 mM formic acid and 100 μL of a 1.8 µg/mL [^13^C_3_]-acrylamide methanolic solution as an internal standard in polypropylene centrifugal tubes and was then stirred for 10 min (Vortex Claver) and centrifuged for another 10 min at 9000 rpm (4 °C) (Sigma 2-16PK). The supernatant was transferred to a 10 mL volumetric flask and the procedure was repeated twice, but with 2.5 mL of 10 mM formic acid. The collected supernatants were treated with 75 µL each of Carrez I (15 g potassium ferrocyanide/100 mL water) and Carrez II (30 g zinc acetate/100 mL water) solutions and centrifuged (9000 rpm for 10 min) at 4 °C to clarify the samples. The supernatant was raised to 10 mL with 10 mM formic acid and cleaned using Oasis-HLB cartridges, preconditioned with 1 mL methanol and 1 mL water. An aliquot of the clear supernatant (1 mL) was loaded onto the cartridge at a flow rate of 2 mL/min; the first 8 drops were discharged and the rest collected. Sample extracts and calibration standards were analysed on an Acquity UHPLC BEH system and a Waters Xevo TQ-S tandem mass spectrometer (Waters Co., Milford, MA). Analytical separation was achieved with an Aquity HPLC HSS T3 (2.1 × 100 mm 1.8 µm). The mobile phase was (A) water and (B) methanol. The binary gradient was 100% A for 2.5 min, 100% B for 2 min and 100% A for 2.5 min, and the flow rate was 0.2 mL/min. The injection volume was 20 µL. 10 mL of standard solutions (1–75μg/L) and was spiked with 100 µL of a 1.8 µg/L [^13^C_3_]-acrylamide methanolic solution for acrylamide quantification. The equation for the curve was Y = 0.858262X + 0.8728 (r^2^ = 0.9952), where Y is the acrylamide peak area/peak area of internal standard ([^13^C_3_]-acrylamide) and X the µg/L of acrylamide. The analyses were conducted in positive ionization mode in multiple-reaction-monitoring (MRM) with the following MRM transitions: 71.9 → 44.0 (quantifier) and 71.9 → 54.5 (qualifier) for acryalmide and 74.0 → 46.1 for [^13^C_3_]-acrylamide. The source temperature was 150 °C, the desolvation temperature was 600 °C, the desolvation gas flow rate was 1200 L/h and the cone gas flow rate was 400 L/h. The LOD and LOQ were 3 and 10 μg/kg, respectively [28].

### 2.5. Furan Assay

The furan analyses were carried out using a modification of the US Food and Drug Administration method [29]. A finely milled sample of 0.5 g was weighed accurately into a 20 mL headspace vial and mixed with 5 mL of saturated sodium chloride solution in milli Q water to which 100 μL of furan-d4 (0.180 μg/mL) were added. The vials were then sealed. Throughout the process, the vials were kept in ice and at a working temperature of 4 °C in order to avoid furan losses. Furan content was determined by headspace extraction gas chromatography mass spectrometry (HS-GC-MS), using a Trace GC Ultra gas chromatograph coupled to a Polaris Q ion-trap mass spectrometer (Thermo Scientific, Barcelona, Spain). The capillary column used was a 30 m × 0.32 mm × 20 µm HP Plot-Q (Agilent Technologies, Santa Clara, CA, USA). The headspace incubation oven temperature was 40 °C and that of the syringe needle, 100 °C. The samples were incubated for 15 min with simultaneous shaking. The injection volume was 2 mL and the injection speed 30 mL/min. The injector temperature was 200 °C with injection in the split mode (split ratio 1:6). Helium was used as a carrier gas, with a flow rate of 1.7 mL/min. The oven temperature was programmed to rise from 50 to 210 °C at 10 °C/min and then held for 10 min. The mass spectrometer was operated in electron impact ionisation mode using automatic gain control with 70 eV of electron energy and 250 mA of emission current. The ion source and transfer line temperatures were 230 °C and 225 °C, respectively. Xcalibur version 2.0.7 software was used for control, general operation and data acquisition. Furan was detected by selective ion monitoring of the major ion at *m*/*z* 68 and confirmed by monitoring the ion at *m*/*z* 39. Furan-d4 was detected by monitoring the equivalent ions at *m*/*z* 72 and *m*/*z* 42. The ion ratio (*m*/*z* 68/39) of the samples was compared and used for further confirmation of a positive identification of furan. 5 mL of standard solutions (1–125 μg/L) were spiked with 100 μL of furan-d4 (0.180 μg/mL) for furan quantification. The equation for the curve was Y = 0.33993X −0.86477 (r^2^ = 0.9980), where Y is the furan peak area/peak area of internal standard (furan-d4) and X the µg/L of furan. The LOD and LOQ were 1.2 μg/kg and 3.8 μg/kg, respectively [10].

### 2.6. Other Determinations

Colour parameters L*, a* and b* were measured using a colourimeter (Model D-25, HunterLab, Reston, VA, USA) following the methodology described by Rodriguez et al. [2]. Moisture, crude protein (N × 6.25), ash content and pH were determined according to official AOAC methods (numbers 970.20, 955.04, 972.15 and 970.21, respectively) [30].

### 2.7. Statistical Analysis

All analyses were performed in duplicate, on duplicate samples (*n* = 4). The results are expressed as mean ± standard deviation (SD). The Shapiro–Wilk test was applied to determine the normality of the variables analysed. The relationships between the different assays were evaluated by computing the corresponding correlation coefficient (Pearson’s linear correlation) at a confidence level of *p* < 0.05. Differences between samples given different thermic treatments were compared by analysis of the variance (ANOVA), at a confidence level of *p* < 0.05. All statistical analyses were performed using IBM SPSS Statistics for Windows, version 28.0 (SPSS, Inc., Chicago, IL, USA).

## 3. Results and Discussion

Cocoa beans are usually roasted at temperatures of between 100 and 180 °C for times appropriate to the temperature applied. At these temperatures, the Maillard reaction occurs and therefore potentially toxic compounds such as acrylamide and furan are produced.

”Dutching” or alkali treatment is used to reduce the bitterness, increase the pH and darken the colour of the cocoa beans. In our analysis, sodium bicarbonate and potassium carbonate were used as alkalising agents, producing average ash contents of 3.9% and 4.5% for the Chuao and Barlovento samples, respectively (Table 2 and Table 3). This content increased according to the concentration of the alkalising agent used, and was higher with potassium carbonate than with sodium bicarbonate. These values are similar to those obtained by Rodríguez et al. [2] and are below the threshold level of 6.5% allowed by the Venezuelan standard COVENIN 1470:1998. According to Rodríguez et al. [2], the resulting pH increases according to the concentration of alkali used (Table 2 and Table 3).

The mean L* (luminosity) values obtained for the Chuao and Barlovento alkalinised samples were 22.1 and 23.1, respectively. L* decreased significantly (*p* < 0.05) (greater darkness) when the concentration of potassium carbonate was increased. Rodríguez et al. [2] analysed alkalised cocoa beans from the Chuao region and reported similar results. However, the colour levels obtained were lower than those found in the present study. Moreover, although the alkalisation conditions applied were similar, Rodríguez et al. [2] used a different heating pattern (150 °C for 30 min).

### 3.1. Furosine

Table 2 and Table 3 show the furosine contents of the cocoa beans from Chuao and Barlovento, respectively. The data were obtained after fermentation and sun-drying, after roasting at 110 °C or 140 °C and after alkalisation with sodium bicarbonate or potassium carbonate at the four concentrations specified. The results obtained for the shells, after separation from the beans, and for each of the treatments specified, are also shown.

The fermented and sun-dried deshelled samples presented the highest values of furosine (Table 2 and Table 3). In cocoa beans, this furosine is generated from the Amadori compound, N-Ɛ- fructoselysine, an adduct of glucose to free lysine of proteins and oligopeptides.

Sucrose metabolises to glucose and fructose during fermentation [31], while free peptides and amino acids increase [32]. In short, fermentation increases the quantity of Maillard reaction reagents produced. Moreover, it raises the temperature of cocoa beans by almost 50 °C, especially during the first 72 h, due to the exothermic reactions associated with aeration and the increased microbial activity [33]. Subsequent drying (30–50 °C) reduces the water content, providing ideal conditions for the generation of Amadori compounds.

The furosine contents in unroasted shells were very high in the beans from both Chuao and Barlovento, but lower than in the nibs, probably due to the lower content of soluble sugars and of lysine, although the carbohydrate and protein contents were higher in unroasted cocoa shells than in unroasted cocoa nibs [34].

When the Chuao cocoa beans were roasted at 110 °C, the furosine content fell by 48%. After alkalisation, this loss was even greater (54–61% for a concentration of 1%) and over 80% at concentrations ≥1.5%, for both alkalising agents. Roasting at 140 °C also reduced furosine levels by approximately 48%, and subsequent alkalisation increased this loss to nearly 83% at a concentration of 1% and to over 90% at concentrations ≥1.5%. Statistical differences (*p* < 0.05) were found between the samples according to the temperature and the degree of alkalisation. When the Chuao samples were roasted at 110 °C, these differences were apparent between different concentrations of the alkalising agent, but not between different agents at a given temperature. A similar trend was obtained for a roasting temperature of 140 °C, except at alkali concentrations of 1.5% and 2.5%. Similar results were also obtained for the Barlovento samples, except when roasting at 140 °C. In summary, therefore, the furosine behaviour of cocoa beans varied according to variety, the roasting temperature and the concentration and temperature of the alkalising agent.

The Maillard reaction continues during roasting. In this respect, Adeyeye et al. [35] analysed the amino acid content of fermented and dried cocoa beans subjected to various thermal processes, including microwave heating to 90–100 °C for 15 min, roasting at 90–100 °C for 20 min, followed by refining to obtain the cocoa liquor and a final heat treatment of 80–90 °C for 12 h. Analysis revealed losses of 90.9% for valine, 78.7% for lysine and 62.4% for histidine, which the authors attributed to the formation of L-amino acid and to the Maillard reaction. Taş and Gökmen [24] observed lower levels of reducing sugars after fermented and dried cocoa beans were roasted at 135 °C and 150 °C for 30 and 60 min. These losses were greater when the fermented and dried cocoa beans were previously immersed in water with sodium carbonate 7.5% for 30 min. Similarly, the losses of lysine in proteins were greater with alkalisation. The authors also reported that the furosine concentration in fermented and dried cocoa beans decreased after roasting at 135 °C and 150 °C and that fructoselysine was more strongly degraded at 150 °C. However, no such differences were observed between alkali-treated and non-treated cocoa beans after roasting.

In our study, the furosine content, at a given temperature (110 °C or 140 °C) was lower in shells than in nibs, probably due to the high impact of the temperature on the outer layers and/or the low concentration of precursors. Furosine losses ranged from 67% after roasting at 110 °C (for the Chuao bean shells) to 93% after roasting at 140 °C (also for the Chuao bean shells). For both types of beans (Chuao and Barlovento) there were significant differences (*p* < 0.05) in the furosine content between nibs and shells, and between fermented, sun-dried, roasted nibs at 110 °C and 140 °C and the corresponding shells.

The mean furosine values (nibs and shells) were 49.0 mg/100 g of protein for the Chuao samples and 103.2 mg/100 g of protein for the Barlovento samples. This difference was statistically significant (*p* < 0.05), and may have been caused by the varying amounts of precursors generated in the fermentation and drying stages, due to different environmental conditions suffered, the artisanal nature of this process and/or the specific characteristics of the variety used (Criollo vs. Trinitario). Although these mean values differed, there was a statistically significant correlation (r = 0.663, *p* < 0.05) between the Chuao and Barlovento samples for the different stages of treatment (fermentation and sun-drying, roasting at 110 °C and 140 °C and alkalisation with sodium bicarbonate or potassium carbonate at concentrations of 1%, 1.5%, 2% and 2.5%), which suggests that this indicator could be used to control the processing of cocoa beans regardless of their origin.

#### Effect of Over-Roasting on Furosine Content

The sustained application of higher temperatures that are habitually used in roasting can reveal the behaviour and characteristics of certain compounds in cocoa beans. In a study of this question, Hinneh et al. [4] roasted forastero cocoa beans at 160 °C for 35 min to determine their aroma profiles.

In our study, the furosine values obtained in the nibs of Barlovento beans, after roasting at 160 °C and 180 °C (Table 4) showed that high temperatures and very short roasting times (15 min) are necessary to obtain high concentrations of Amadori compounds, although values < 9 mg of furosine/100 g of protein are obtained after 30 min of treatment. Alkalisation provokes a major loss of furosine (76%), similar to that obtained after roasting at 110 °C and 140 °C. There were statistically significant differences (*p* < 0.05) between the untreated samples and those roasted at 160 °C and 180 °C.

These extreme treatments show that for each temperature there is a critical moment at which Amadori compounds are destroyed faster than they are generated. For example, when the beans are roasted for 30 min at 180 °C or for 40 min at 160 °C, the Amadori compounds are almost completely destroyed; however, at a temperature of 140 °C, even one hour of roasting does not completely eliminate the compounds.

**Figure 1:**
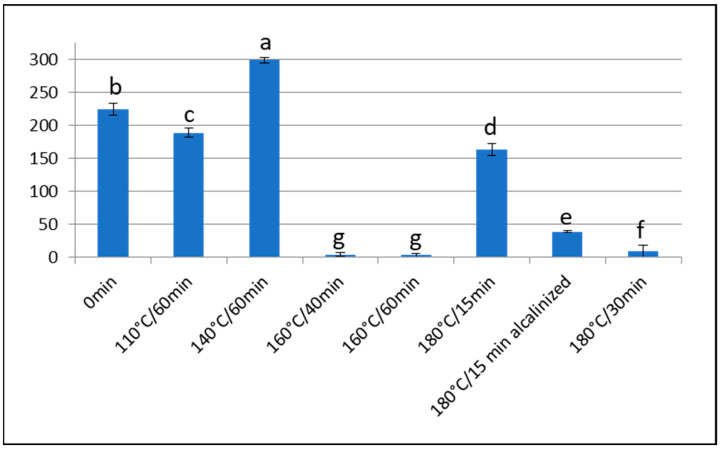
Furosine content (mg/100 g of protein) of Barlovento cacao samples subjected to different types of processing. Different letters represent significant differences (*p* < 0.05).

To our knowledge, this study is only the second to consider furosine content as a useful indicator during thermal control of the different stages of cocoa processing. The furosine content recorded after extreme heat treatment corroborates the usefulness of this indicator for controlling processes such as fermentation, drying and roasting, whether at sustained, relatively low temperatures or at high temperatures and shorter durations, as well as the subsequent effects of alkalisation (Figure 1). The importance of this consideration is that controlling fermentation during the post-harvest stage of cocoa bean processing is crucial for the proper formation of aromatic compounds [5].

### 3.2. Acrylamide

As expected, the fermented, sun-dried samples (nibs and shells) from Chuao and Barlovento did not present detectable levels of acrylamide. However, this compound was generated during roasting. At 110 °C, acrylamide contents of 222 µg/kg and 154 µg/kg were found in Chuao and Barlovento nibs, respectively, and these levels were significantly higher (*p* < 0.05) at 140 °C (Table 2 and Table 3).

The acrylamide content in the roasted shells was higher than in the nibs, possibly because the carbohydrate and protein contents are higher in roasted shells than in roasted nibs [34]. The difference was statistically significant (*p* < 0.05) in both regions and at both temperatures, except at 110 °C for the Chuao samples. Similar data have been reported by other studies. Thus, Barišić et al. [36] measured 166 µg/kg of acrylamide in cocoa shells obtained after roasting whole beans at 135 °C for 55 min, and Żyżelewicz et al. [37] found a higher acrylamide content in whole rather than in deshelled cocoa beans after roasting.

Research findings on the presence of acrylamide in cocoa vary greatly; the FAO/WH [12] reported an average acrylamide content of 220 µg/kg with a maximum value of 909 µg/kg. In our study, similar values were found in cocoa beans roasted at 110 °C. However, the Barlovento shells roasted at 110 °C and 140 °C and the Chuao shells roasted at 140 °C, presented values above the maximum value indicated by the FAO/WHO [12]. In addition, Granvogl and Schieberle [13] found values ranging from 63 to 643 µg/kg, while Pardo et al. [13] obtained average values of 102 µg/kg in 15 samples of chocolate purchased from supermarkets in Valencia (Spain). Arisseto et al. [15] reported acrylamide values of 67 µg/kg for cocoa powder and 150 µg/kg for chocolate. Köppen et al. [16] analysed 140 samples (chocolates with various levels of cocoa and cocoa powders) sourced from German retail markets, and measured acrylamide levels of 9–1747 μg/kg. The EU database for acrylamide [17] records a mean acrylamide level of 178 μg/kg for 13 cocoa powder samples. Finally, Raters and Matissek [18] analysed commercial semi-finished cocoa products (cocoa beans, nibs, powder, masses and cocoa butter) and found acrylamide in 97% of the samples examined, with 61% presenting acrylamide levels ranging from 101 to 400 μg/kg. The mean values for nibs was 200 μg/kg.

The effect of cocoa processing on acrylamide content has also received considerable research attention. Some authors have detected acrylamide in unroasted cocoa beans. For example, Granvogl and Schieberle [13] found acrylamide in 7-day fermented and sun-dried cocoa beans obtained from Ghana and Indonesia (64.3 and 79.2 μg/kg, respectively), while Raters and Matissek [18] reported similar results for unroasted cocoa beans of unknown origin (50−80 µg/kg). Kruszewski and Obiedziński [7] found acrylamide levels of 48.2 and 32.3 μg/kg in fermented, dried cocoa beans from Ecuador and Dominican Republic, respectively. However, other authors, such as Farah et al. [19] and Żyżelewicz et al. [37] did not detect any acrylamide in fermented, dried cocoa beans, and Gil et al. [20] in their analysis of two samples of fermented, dried cocoa beans from two sub-regions of Antioquia-Colombia (Bajo Cauca and Magdalena Media) found acrylamide (79 µg/kg) in the first, but not in the second. This discrepancy might be attributed to differences in the fermentation methods (time, temperature and the microorganism involved), and/or in the drying conditions (time, temperature and kind of vessels) and/or the characteristics of the geographical origins of the cocoa beans [7]. Granvogl and Schieberle [13] suggested that the fermentation process generates 3-aminopropionamide, as well as specific conditions (such as low pH) that may facilitate the formation of acrylamide.

During roasting, the content of acrylamide increases. Granvogl and Schieberle [13] analysed non-fermented and fermented sun-dried cocoa samples from Ghana and Indonesia, before and after roasting at 95 °C for 15 min. This temperature was subsequently increased by 1 °C/min up to 115 °C, which was maintained for 20 min. The following acrylamide values were found: for non-fermented and fermented Ghana beans, 223 and 922 µg/kg, respectively; for the Indonesian beans, 122 and 337 µg/kg for the non-fermented and fermented samples, respectively. Farah et al. [19] analysed the acrylamide content in deshelled cocoa beans from Cameroon, Indonesia, Malaysia, Ivory Coast and Papua New Guinea, roasted at 116 °C for 23 min, finding values of 1100, 1300, 1700, 2300 and 3200 µg/kg, respectively. When the Indonesia beans were roasted at different temperatures for 25 min, the values found, 1200 and 7800 µg/kg at 110 °C and 150 °C, respectively, were higher than those measured in the present study. This difference might be due to the fact that the beans were roasted without the shell or because the authors determined the acrylamide content by gas chromatography and flame ionisation detection. This technique is less selective than mass spectrometry and therefore might have led to an overestimation, highlighting the fact that cocoa is one of the most challenging food matrices for acrylamide analysis due to interferences [38,39]. Żyzelewicz et al. [37] showed that the roasting of whole cocoa beans of the forastero variety (from Togo) at 135 and 150 °C, but at different relative humidities (RH) in the air (dry, RH = 0.3 vs. humid RH = 5%), produced significantly different rates of acrylamide formation, ranging from 62 to 150 μg/kg. The highest content was produced at 135 °C (RH = 5%); at this temperature, a longer roasting time (50 min) was needed to achieve the necessary water content (2% *w*/*w*). When deshelled beans were roasted, lower levels of acrylamide were obtained, probably because less processing time was required to obtain the final product. Finally, Kruszewski and Obiedziński [7] found lower concentrations of acrylamide, with average contents of 108.0 µg/kg and 93.9 µg/kg for cocoa beans from Ecuador and the Dominican Republic, respectively, when shelled cocoa beans were toasted at 150 °C. The discrepancies observed in the acrylamide content may be due to one or more of the following factors: (1) the variety and geographical origin of the cocoa beans; (2) prevailing agro-ecological conditions and the post-harvest treatment applied; (3) the form of the beans, i.e., whole beans or nibs; (4) the roasting conditions (temperature, RH and time) applied. Although the samples from Barlovento (“trinitario”) and Chuao (“criollo”) were processed in similar ways, the precursor contents probably favoured the greater generation of acrylamide (in nibs and shells at 110 °C and 140 °C) in the Barlovento beans.

In November 2019, the European Union first recommended monitoring the presence of acrylamide in roasted cocoa beans and derived cocoa products [40]. Although the content of acrylamide in cocoa is not currently regulated, it is significant that in our analysis, Chuao beans roasted at 140 °C/1h exceeded the reference level established by the Commission regulation of the European Union [41] for the reduction of the presence of acrylamide in roasted coffee (400 µg/kg).

The alkalisation and subsequent drying of the Chuao and Barlovento samples produced significant statistical differences (*p* < 0.05) between the nibs roasted at 110 °C and 140 °C and those subjected to alkalisation with sodium bicarbonate or potassium carbonate at 1% and 2.5%. Differences were also observed according to the concentration of alkali used (1% vs. 2.5%), for both alkalis.

Each of the samples roasted at 140 °C presented a similar response to alkalisation; the initial content of acrylamide was high (Table 2 and Table 3); this level decreased when the beans were treated with sodium bicarbonate and increased with potassium carbonate. In the Chuao beans roasted at 110 °C, acrylamide levels fell when the samples were alkalised with sodium bicarbonate or potassium carbonate except for those subjected to 2.5% NaHC0_3_. In the Barlovento samples, however, acrylamide levels rose for the two alkalising agents except when 2.5% K_2_CO_3_ was applied.

Surprisingly, after alkalisation and drying, the moisture of the samples presented a positive relation with the acrylamide content (Table 2 and Table 3). Moreover, the average level of acrylamide in the samples treated with potassium carbonate was higher than in those treated with sodium bicarbonate, and there was a greater acrylamide content at a higher pH. Basic pH favours the formation of reducing sugars in the open configuration and amino group deproteinisation, which in turn facilitates the Maillard reaction. In this respect, Huang and Barringer [42] reported that peak concentrations of pyrazines and Strecker compounds were detected at shorter times when cocoa beans roasted at a given temperature were alkalised with potassium carbonate.

The generation of acrylamide during alkalisation depends on the drying temperature applied and on the pH of the process. At 140 °C, a much larger amount of acrylamide was generated (Table 2 and Table 3), and probably very few precursors remained. The application of heat during the drying stage does not favour the formation of acrylamide unless the pH is very basic, as would be achieved with potassium carbonate. After alkalisation, drying temperatures similar to those reached during fermentation can also generate acrylamide.

Taeymans et al. [43] reported that alkalisation produced varying effects on acrylamide levels, while Kruszewski and Obiedziński [7] found similar acrylamide contents in two alkalised samples that had previously differed (although the type and concentration of alkali used were not stated). In a study by Ofosu et al. [9], deshelled Ghana cocoa beans were alkalised with different solutions of K_2_CO_3_ at concentrations from 10 to 70% *w*/*v*. The nibs were then roasted at temperatures of 110 °C and 140 °C for 20 and 50 min, respectively, to optimise the roasting-time cycle of the alkalisation and thus minimise the formation of acrylamide. After 23 experiments, the optimal conditions were found to be roasting at 110 °C for 20 min and alkalisation with 29.2% K_2_CO_3_. In most of the experiments, when alkali was applied before roasting, the acrylamide content increased. Variations in the concentration of potassium carbonate also affected acrylamide levels. These authors concluded that immersion in an alkaline medium accelerated the Maillard reaction and favoured the formation of acrylamide.

In our study, sodium bicarbonate should be used as an alkalising agent in order not to exceed the value of 400 μg/kg established for roasted coffee as a maximum reference value [41].

Although average acrylamide levels differed between Chuao and Barlovento beans, analysis revealed a correlation of r = 0.518 (*p* < 0.05) between the various stages of processing (fermentation and sun-drying, roasting at 110 °C or 140 °C and the alkalisation of the roasted beans with sodium bicarbonate or potassium carbonate at concentrations of 1% or 2.5%), indicating that these different treatments have comparable outcomes as regards the generation of acrylamide in the beans from both regions.

#### Effect of Over-Roasting on Acrylamide Content

**Figure 2:**
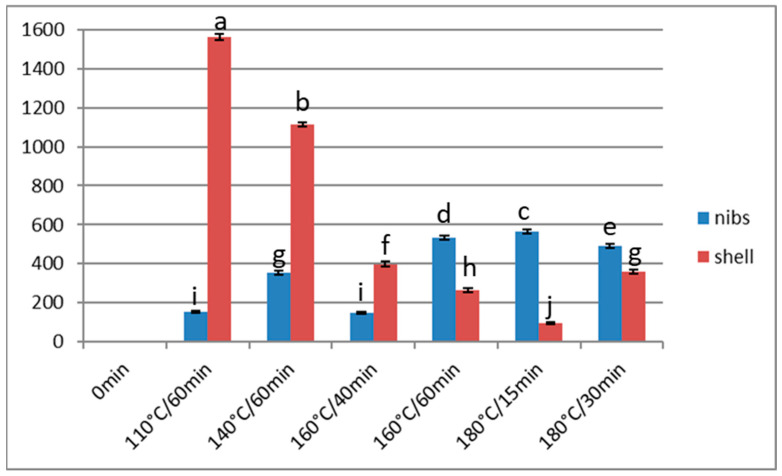
Acrylamide content (μg/kg) of Barlovento nibs and shells subjected to different types of processing. Different letters represent significant differences (*p* < 0.05).

When Barlovento cocoa beans were roasted at 160 °C, the acrylamide content of the nibs increased significantly, from 146 µg/kg at 40 min to 532 µg/kg at 60 min (*p* < 0.05). The latter value is higher than those obtained for roasted nibs at 110 °C and 140 °C. At 180 °C, the acrylamide content decreased inversely to the roasting time, with a reduction of 72 µg/kg during the interval from 15 to 30 min. On the other hand, the value obtained at 180 °C after 15 min of roasting was slightly higher than that obtained at 160 °C after 60 min (Figure 2).

The response of the acrylamide content in the shells differed according to the temperature applied (110 °C or 140 °C vs. 160 °C). At the less extreme treatments (up to 160 °C/40 min), the acrylamide content in the shells was higher than in the nibs, but with more drastic treatments, the situation was reversed. At very high temperatures, the acrylamide is degraded, and this effect is more appreciable in the shell, where the temperature impact is greatest (Figure 2).

In the Barlovento samples, temperatures above 180 °C for 30 min were sufficient to initiate the destruction of acrylamide. Farah et al. [19] determined acrylamide contents in fermented and roasted cocoa samples heated to 130 °C for various times up to 40 min, and recorded a maximum value of 7800 µg/kg at 30 min, which decreased to 7300 µg/kg at 40 min. These authors also determined the acrylamide content for a constant time (25 min) at roasting temperatures from 100 °C to 160 °C, obtaining a maximum acrylamide value at 150 °C (7800 µg kg), which decreased to 7200 µg/kg at 160 °C.

As mentioned above, in the study by Żyżelewicz et al. [37], the acrylamide content obtained when whole cocoa beans were roasted at 150 °C (RH = 5%) was significantly lower than that obtained when the roasting was performed at 135 °C, with the same RH. The authors suggested that this compound might be lost by volatilisation, among other reasons. However, the duration of each treatment also differed (50 min at 135 °C and 35 min at 150 °C). According to our data, for acrylamide to be volatilised faster than it is generated in the nibs, the temperature applied must exceed 150 °C.

**Figure 3:**
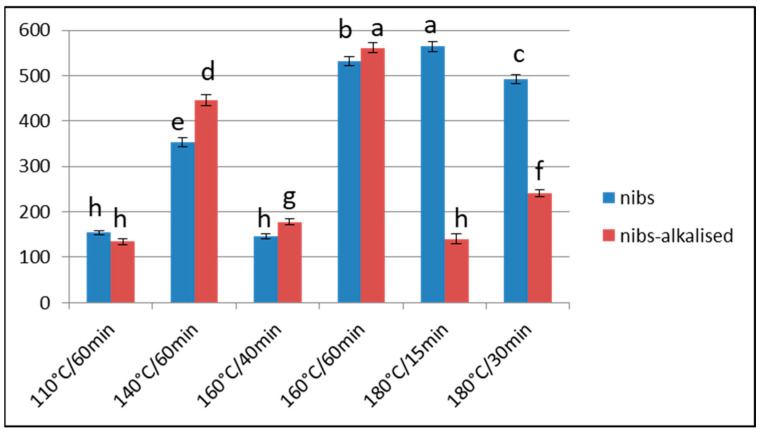
Acrylamide content (μg/kg) of Barlovento nibs alkalised (with K_2_CO_3_) or otherwise when subjected to different types of processing. Alkali concentration: 2.5% at 110 °C or 140 °C, 2% at 160 °C or 180 °C. Different letters represent significant differences (*p* < 0.05).

In the extreme treatments applied in our study, the alkalisation was only carried out with 2% potassium carbonate, in order to obtain a high pH value in the nibs. At 160 °C, the acrylamide content was significantly greater in the alkaline nibs (*p* < 0.05), but this situation was reversed at 180 °C. These data are in line with those obtained for less extreme treatments. Thus, the acrylamide content is higher when the roasting is performed at 110 °C and 140 °C, or at 160 °C for 1 h, and the pH is more alkaline. In contrast, it is lower when the pH is less basic (when alkalisation is achieved by sodium bicarbonate) and when the roasting treatment is extreme (180 °C). Under this latter condition, there will probably be no precursors and the degradation of acrylamide will exceed its generation (Figure 3).

In this study, only treatment at 160 °C for 40 min with or without alkalisation produced acrylamide levels lower than 400 μg/Kg.

### 3.3. Furan

The fermented, sun-dried, deshelled cocoa samples from the Chuao region had a furan content of 7.6 µg/kg; this value increased to 18 µg/kg after roasting at 110 °C and to 46 µg/kg at 140 °C. In both cases, the difference was statistically significant (*p* < 0.05). The furan content in the fermented and sun-dried samples from Barlovento was slightly higher (9.3 µg/kg) and the only significant increase (to 23 µg/kg; *p* < 0.05) was observed after roasting at 140 °C (Table 2 and Table 3).

The furan contents in the roasted shells at 140 °C (Chuao samples) and at 110 °C (Barlovento samples) were higher than in the nibs (*p* < 0.05). Unlike acrylamide, furan is a very volatile compound and at low temperatures, the rate of generation is probably not high. In consequence, its elimination is favoured, which would explain the different response patterns observed between the shells and the nibs at the same roasting temperatures.

To date, few studies have addressed the question of furan content in cocoa products. The US Department of Food and Drug Administration [21] reported values of <0.4 to 10.3 µg/kg in mixtures of chocolate drinks, cocoa, chocolate syrups and chocolate drinks. The European Food Safety Agency [22] reported the furan content of 14 samples, with average values of 9–10 µg/kg, median values of 5.4–6 µg/kg and a maximum value of 40 µg/kg, but did not specify which cocoa products were analysed. The UK Food Standard Agency [23] reported values between 12 and 20 µg/kg in six commercial samples of cocoa and chocolate powder. In a recent study, Kruszewski and Obiedziński [7] reported high levels of furan (25.1 and 34.8 µg/kg) in roasted cocoa beans from Ecuador and the Dominican Republic, respectively.

In our analysis, the furan content statistically decreased (*p* < 0.05) after alkalisation with both alkalis. The greatest decreases were obtained at high roasting temperatures and when potassium carbonate was applied. For both varieties, the average losses at 110 °C were 31–37% with potassium carbonate, 57–60% with sodium bicarbonate and 71–76% with potassium carbonate at 140 °C (Table 2 and Table 3). Statistically significant differences were not always observed between the treatments involving the same alkali at different concentrations; however, there were almost always differences between those in which the concentration remained unchanged but different alkalis (bicarbonate or carbonate) were used (*p* < 0.05). Kruszewski and Obiedziński [7] reported furan contents of 10.2 and 3.3 µg/kg in two samples of cocoa powder with different degrees of alkalisation; the less alkalised samples had three times more furan than the most strongly alkalised ones.

There was a significant inverse correlation between pH and furan content in the alkalised samples, ranging from r = −0.6934 to −0.8615 (*p* < 0.05). According to Nie et al. [44], the degradation of fructose (the majority reducing sugar in cocoa) is greater at neutral pH and that of glucose is favoured at basic pH. These findings could explain the different responses of furan and acrylamide to alkalisation. In our results, a significant inverse relation was observed between the final moisture of the alkalised cocoa nibs and the furan content, ranging from r = −0.3186 for the Chuao samples at 110 °C to r = −0.8604 for the Barlovento ones at 140 °C. Due to the volatility and low drying temperatures of alkalisation, more furan is eliminated than is generated. Moreover, the small amount that may be formed (since fermented, dried grains contain furan) is reduced in the presence of a more basic pH. A small non-significant negative relationship was recorded between the browning index and the furan content in the alkalised samples (Table 2 and Table 3). Thus, the stronger the Maillard reaction, the more intense the colour and the lower the furan content, due to volatilisation. To our knowledge, no previous studies have been undertaken to determine the presence of furan when roasted cocoa beans are subjected to alkalisation.

The mean furan contents in the samples analysed (both alkalised and non-alkalised) was 18.7 µg/kg for the Chuao samples and 9.1 µg/kg for the Barlovento samples. This difference was statistically significant (*p* < 0.05). In contrast to acrylamide, the furan content in the Chuao samples was higher than in those from Barlovento. It is important to note that furan can be generated by different pathways, for example, through the degradation of polyunsaturated fatty acids and carotenoids, and not only by the Maillard reaction (affecting amino acids and sugars); moreover, in the Maillard reaction, amino groups other than asparagine may participate. These considerations could account for the differences observed in our data. The correlation between the Chuao and Barlovento samples for the different conditions analysed (fermentation and sun-drying, roasting at 110 °C and 140 °C and alkalisation of the roasted nibs with either sodium bicarbonate or potassium carbonate at concentrations ranging from 1% to 2.5%) was r = 0.672 (*p* = < 0.05). The different treatments applied had an equal degree of impact on the generation of furan in the cocoa samples from both varieties.

#### Effect of Over-Roasting on Furan Content

**Figure 4:**
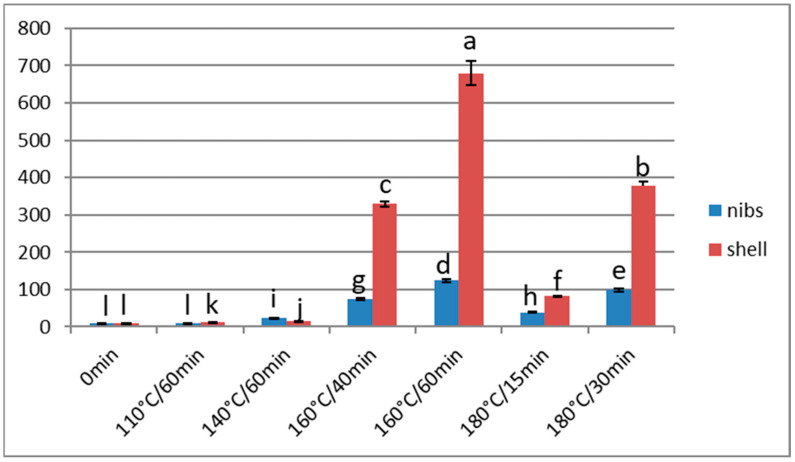
Furan content (μg/kg) of Barlovento nibs and shells subjected to different types of processing. Different letters represent significant differences (*p* < 0.05).

In the samples analysed, the furan content increased significantly (*p* < 0.05) when the roasting temperature exceeded 160 °C. At 180 °C and a time of 30 min, the content was higher than at 160 °C for 40 min. Moreover, the content in the shells was higher than in the nibs, and this difference increased with time, for a given temperature. Thus, the furan contents were 4.4 and 5.5 times higher when the beans were roasted at 160 °C for 40 min and 60 min, respectively. At 180 °C, the relation between the furan content in the shells and in the nibs was 2.1 and 3.8-fold, for times of 15 and 30 min, respectively (Figure 4). At both temperatures, this difference was statistically significant (*p* < 0.05). Furthermore, and as noted above, furan can be generated in food by several pathways, via the individual degradation of amino acids and sugars, through the Maillard reaction or via the degradation of ascorbic acid and polyunsaturated fatty acids. The latter two precursors have little or no presence in the shells, and so the Maillard reaction and the individual degradation of sugars and amino acids would probably be the main channels for the generation of this compound. Agus et al. [34] measured higher carbohydrate and protein contents in roasted cocoa shells than in roasted cacao nibs. Moreover, the formation in shells is favoured by the fact that the temperature is generally higher on the outer part of the grain.

The furan values obtained when the nibs were roasted at 160 °C for 1 h were much higher than those obtained at 110° or 140 °C for the same processing time (Figure 4). From this, we conclude that extremely high temperatures (i.e., 160 °C and 180 °C) should not be used unless the roasting time is decreased correspondingly.

**Figure 5:**
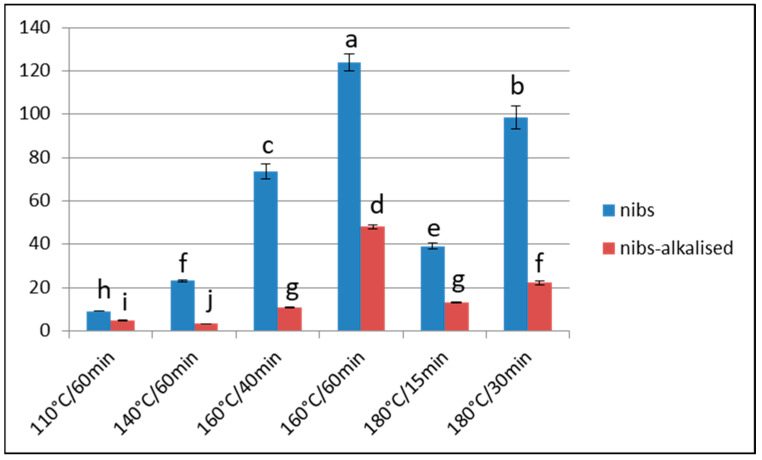
Furan content (μg/kg) of Barlovento nibs, alkalised (with K_2_CO_3_ at 2%) or otherwise, when subjected to different types of processing. Different letters represent significant differences (*p* < 0.05).

For nibs roasted at 160 °C or 180 °C, alkalisation with 2% potassium carbonate resulted in statistically significant decreases (*p* < 0.05) in the furan content, of 61% and 83%, respectively (Table 4). This response is probably due either to the creation of a basic pH environment, or to the greater elimination rather than generation of furan at the low drying temperatures used after alkalisation (or to a combination of these factors). Similar results were obtained for the samples roasted at 110 °C and 140 °C (Figure 5).

For both of these toxic compounds (acrylamide and furan), treatment at 140 °C for 1 h should not be exceeded in order to ensure that the values in the nibs remain below the limits established for similar foods, such as coffee. When the shells are used as a by-product in functional foods [45], this temperature would lead to the above limits being exceeded.

### 3.4. Correlations between Furosine, Furan and Acrylamide

#### 3.4.1. Correlations between Acrylamide and Furan

A positive relation was observed between acrylamide and furan when the nibs from Chuao or Barlovento were roasted at 110 °C or 140 °C, with r = 0.9719 (*p* < 0.05), and with r = 0.5875 if the data for roasting temperatures of 160 °C and 180 °C are included.

#### 3.4.2. Correlations between Furosine and Acrylamide/Furan

For the Chuao nibs and shells, the correlation obtained between furosine and acrylamide for roasting at 110 °C and 140 °C was r = −0.6981 (*p* < 0.05). For the Barlovento samples, the correlation was r = −0.8093 (*p* < 0.05). With alkalisation, the relation was negative, but was only statistically significant in the Barlovento samples that were roasted at 140 °C (r = −0.6927).

For the Chuao beans, the correlation obtained between furosine and furan in the roasting treatments (110 °C and 140 °C) was r = −0.7063 for the nibs and r = −0.8304 for the shells. For the Barlovento samples, the relationship was r = −0.8833 (110–180 °C) for the nibs and r = −0.6745 for the shells. During alkalisation, the trend was positive for the nibs from Chuao (r = 0.3865) and from Barlovento (r = 0.4563). The different behaviour of furan and acrylamide is probably due to the volatility of furan.

#### 3.4.3. Other Correlations

*Moisture in the alkalised samples*. The correlations between furosine and moisture in alkalised Chuao and Barlovento beans were negative and statistically significant (*p* < 0.05), with r = −0.3216 and r = −0.2536, respectively. We also obtained negative correlations for furan in the same samples, with r = −0.4308 and r = −0.7680. However, positive and non-logical correlations were obtained with acrylamide, which suggests that the alkalising agent or pH obtained plays a stronger role in the generation/destruction of this compound during alkalisation than the final moisture content, which indicates the intensity of the drying process.

*Browning index (BI)*. The Browning index was calculated from the colour of non-roasted cocoa nibs and of alkalised nibs after roasting. The Barlovento and Chuao samples presented similar increases in mean BI, of 16.08 and 15.9, respectively. The index values for the samples roasted and alkalised at 110 °C were higher than those for the samples roasted and alkalised at 140 °C, probably because some of the compounds responsible for the colour are destroyed at high temperatures, a loss that is not compensated by the compounds generated by the Maillard reaction.

A strong relation was observed between BI and pH, especially in the Barlovento samples (r = 0.9936). A slight negative relation was obtained between BI and furosine in the roasted and alkalised samples from Chuao (r = −0.2473) and from Barlovento (r = −0.3323), but no such relation was obtained between BI and furan.

## 4. Conclusions

Furosine is a useful indicator of the acrylamide content of cocoa beans processed at moderate temperatures (≤140 °C), especially in the fermentation, drying and roasting stages. The acrylamide content of the nibs increases in line with the roasting temperature to 160 °C, while in the shells it may be destroyed at lower temperatures. The acrylamide content in the shells at normal roasting temperatures is higher than in the nibs. The furan content in the nibs increases with the roasting temperature, becoming noticeable at temperatures of 140 °C and very high at temperatures ≥160 °C. In this case too, the content is greater in the shells than in the nibs. The high content of acrylamide and furan in the roasted shell means that this by-product should not be included in foods. In all cases, alkalisation after roasting reduces the furan content but not that of acrylamide. In this respect, sodium bicarbonate generally has a stronger effect than potassium carbonate. The response patterns of furosine, acrylamide and furan during roasting and alkalisation were similar in the beans from each of the varieties analysed. To minimise the generation of acrylamide and furan, both of which are toxic compounds, the optimum approach to artisanal cocoa processing is treatment at 110 °C for 1 h and alkalisation with sodium bicarbonate at 10 g/L. Moreover, the fermentation process could be controlled with furosine to further reduce the presence of toxic compounds in the production of cocoa.

## Figures and Tables

**Table 1:** Coding for treatments applied to cacao beans from Chuao and Barlovento (Venezuela).

Code	Sample	Code	Sample
NC0	Nibs Chuao control	NB0	Nibs Barlovento control
SC0	Shell Chuao control	SB0	Shell Barlovento control
NC110	Nibs Chuao roasted 110 °C (60 min)	NB110	Nibs Barlovento roasted 110 °C (60 min)
SC110	Shell Chuao roasted 110 °C (60 min)	SB110	Shell Barlovento roasted 110 °C (60 min)
NC140	Nibs Chuao roasted 140 °C (60 min)	NB140	Nibs Barlovento roasted 140 °C (60 min)
SC140	Shell Chuao roasted 140 °C (60 min)	SB140	Shell Barlovento roasted 140 °C (60 min)
NC110Na10	Nibs Chuao roasted 110 °C (60 min) NaHCO_3_ 10 g kg^−1^	NB110Na10	Nibs Barlovento roasted 110 °C (60 min) NaHCO_3_ 10 g kg^−1^
NC110Na15	Nibs Chuao roasted 110 °C (60 min) NaHCO_3_ 15 g kg^−1^	NB110Na15	Nibs Barlovento roasted 110 °C (60 min) NaHCO_3_ 15 g kg^−1^
NC110Na20	Nibs Chuao roasted 110 °C (60 min) NaHCO_3_ 20 g kg^−1^	NB110Na20	Nibs Barlovento roasted 110 °C (60 min) NaHCO_3_ 20 g kg^−1^
NC110Na25	Nibs Chuao roasted 110 °C (60 min) NaHCO_3_ 25 g kg^−1^	NB110Na25	Nibs Barlovento roasted 110 °C (60 min) NaHCO_3_ 25 g kg^−1^
NC110K10	Nibs Chuao roasted 110 °C (60 min) K_2_CO_3_ 10 g kg^−1^	NB110K10	Nibs Barlovento roasted 110 °C (60 min) K_2_CO_3_ 10 g kg^−1^
NC110K15	Nibs Chuao roasted 110 °C (60 min) K_2_CO_3_ 15 g kg^−1^	NB110K15	Nibs Barlovento roasted 110 °C (60 min) K_2_CO_3_ 15 g kg^−1^
NC110K20	Nibs Chuao roasted 110 °C (60 min) K_2_CO_3_ 20 g kg^−1^	NB110K20	Nibs Barlovento roasted 110 °C (60 min) K_2_CO_3_ 20 g kg^−1^
NC110K25	Nibs Chuao roasted 110 °C (60 min) K_2_CO_3_ 25 g kg^−1^	NB110K25	Nibs Barlovento roasted 110 °C (60 min) K_2_CO_3_ 25 g kg^−1^
NC140Na10	Nibs Chuao roasted 140 °C (60 min) NaHCO_3_ 10 g kg^−1^	NB140Na10	Nibs Barlovento roasted 140 °C (60 min) NaHCO_3_ 10 g kg^−1^
NC140Na15	Nibs Chuao roasted 140 °C (60 min) NaHCO_3_ 15 g kg^−1^	NB140Na15	Nibs Barlovento roasted 140 °C (60 min) NaHCO_3_ 15 g kg^−1^
NC140Na20	Nibs Chuao roasted 140 °C (60 min) NaHCO_3_ 20 g kg^−1^	NB140Na20	Nibs Barlovento roasted 140 °C (60 min) NaHCO_3_ 20 g kg^−1^
NC140Na25	Nibs Chuao roasted 140 °C (60 min) NaHCO_3_ 25 g kg^−1^	NB140Na25	Nibs Barlovento roasted 140 °C (60 min) NaHCO_3_ 25 g kg^−1^
NC140K10	Nibs Chuao roasted 140 °C (60 min) K_2_CO_3_ 10 g kg^−1^	NB140K10	Nibs Barlovento roasted 140 °C (60 min) K_2_CO_3_ 10 g kg^−1^
NC140K15	Nibs Chuao roasted 140 °C (60 min) K_2_CO_3_ 15 g kg^−1^	NB140K15	Nibs Barlovento roasted 140 °C (60 min) K_2_CO_3_ 15 g kg^−1^
NC140K20	Nibs Chuao roasted 140 °C (60 min) K_2_CO_3_ 20 g kg^−1^	NB140K20	Nibs Barlovento roasted 140 °C (60 min) K_2_CO_3_ 20 g kg^−1^
NC140K25	Nibs Chuao roasted 140 °C (60 min) K_2_CO_3_ 25 g kg^−1^	NB140K25	Nibs Barlovento roasted 140 °C (60 min) K_2_CO_3_ 25 g kg^−1^
NB16040	Nibs Barlovento roasted 160 °C (40 min)	SB16040	Shell Barlovento roasted 160 °C (40 min)
NB16060	Nibs Barlovento roasted 160 °C (60 min)	SB16060	Shell Barlovento roasted 160 °C (60 min)
NB18015	Nibs Barlovento roasted 180 °C (15 min)	SB18015	Shell Barlovento roasted 180 °C (15 min)
NB18030	Nibs Barlovento roasted 180 °C (30 min)	SB18030	Shell Barlovento roasted 180 °C (30 min)
NB16040K20	Nibs Barlovento roasted 160 °C (40 min) K_2_CO_3_ 20 g kg^−1^	NB18015K20	Nibs Barlovento roasted 180 °C (15 min) K_2_CO_3_ 20 g kg^−1^
NB16060K20	Nibs Barlovento roasted 160 °C (60 min) K_2_CO_3_ 20 g kg^−1^	NB18030K20	Nibs Barlovento roasted 180 °C (30 min) K_2_CO_3_ 20 g kg^−1^

**Table 2:** Analysis results for Chuao beans treated at 110 °C and 140 °C ^1^.

Code	Furosine (mg/100 g of Protein)	Acrylamide (µg/kg)	Furan (µg/kg)	Moisture (%)	L	Browning Index (100-L^•^)	pH	Ash (%)
NC0	249 ± 1 ^a^	nd ^j^	7.60 ± 0.99 ^k^	-	29.08 ± 0.02 ^b^	70.92	5.0	-
SC0	151 ± 1 ^b^	nd ^j^	4.18 ± 0.08 ^l^	-	-	-	-	-
NC110	129 ±12 ^c^	222 ± 7 ^g^	17.9 ± 0.5 ^e,f^	-	-	-	-	-
SC110	49.1 ± 3.9 ^d^	204 ± 10 ^g^	16.0 ± 0.4 ^f,g^	-	-	-	-	-
NC140	129 ± 12 ^c^	627 ± 8 ^e^	46.2 ± 1.4 ^b^	-	-	-	-	-
SC140	9.88 ± 0.91 ^g,h,i^	956 ± 12 ^b^	65.2 ± 1.9 ^a^	-	-	-	-	-
NC110Na10	59.6 ± 5.4 ^d^	95 ± 8 ^h^	34.6 ± 1.5 ^c^	4.81 ± 0.21 ^i^	21.60 ± 0.02 ^j^	78.40	6.5	3.19 ± 0.09 ^g^
NC110Na15	22.3 ± 1.9 ^e,f^	-	12.5 ± 0.3 ^h,i,j^	5.15 ± 0.26 ^h,i^	19.10 ± 0.03 ^n^	80.90	7.1	3.85 ± 0.02 ^d,e,f^
NC110Na20	34.3 ± 1.9 ^e^	-	19.8 ± 0.6 ^e^	5.01 ± 0.01 ^i^	23.90 ± 0.02 ^f^	76.10	7.9	4.02 ± 0.03 ^c,d^
NC110Na25	16.5 ± 1.5 ^f,g,h,i^	1253 ± 11 ^a^	13.0 ± 0.4 ^h,i^	7.05 ± 0.05 ^e,f^	17.20 ± 0.02 ^p^	82.80	8.0	4.51 ± 0.01 ^b,c^
NC110K10	51.2 ± 3.9 ^d^	385 ± 12 ^f^	7.31 ± 0.30 ^k^	5.09 ± 0.15 ^h,i^	27.90 ± 0.02 ^c^	72.10	-	4.15 ± 0.38 ^b,c,d^
NC110K15	25.9 ± 2.3 ^e,f^	-	14.7 ± 0.8 ^g,h^	7.58 ± 0.08 ^d^	20.50 ± 0.03 ^k^	79.50	-	4.04 ± 0.33 ^c,d^
NC110K20	19.8 ± 1.3 ^f,g,h^	-	12.6 ± 0.1 ^h,i^	8.56 ± 0.37 ^c^	18.00 ± 0.05 ^o^	82.00	-	4.20 ± 0.36 ^b,c,d^
NC110K25	15.6 ± 1.4 ^f,g,h,i^	25.0 ± 0.8 ^i^	14.6 ± 0.4 ^g,h^	4.81 ± 0.68 ^i^	16.90 ± 0.03 ^q^	83.10	-	3.23 ± 0.19 ^f,g^
NC140Na10	22.8 ± 1.3 ^e,f^	25.0 ± 1.1 ^i^	17.5 ± 0.4 ^e,f^	3.35 ± 0.27 ^j^	25.20 ± 0.04 ^e^	74.80	-	3.16 ± 0.23 ^g^
NC140Na15	12.9 ± 1.3 ^f,g,h,i^	-	18.8 ± 0.4 ^e^	4.44 ± 0.00 ^i^	29.20 ± 0.02 ^a^	70.80	-	2.50 ± 0.08 ^h^
NC140Na20	11.9 ± 1.0 ^g,h,i^	-	14.0 ± 0.2 ^g,h^	4.38 ± 0.07 ^i^	21.90 ± 0.03 ^i^	78.10	-	3.35 ± 0.08 ^e,f,g^
NC140Na25	8.30 ± 0.15 ^g,h,i^	85.0 ± 7.4 ^h^	23.0 ± 0.9 ^d^	4.59 ± 0.32 ^i^	23.10 ± 0.03 ^h^	76.90	-	4.20 ± 0.21 ^b,c,d^
NC140K10	21.8 ± 1.0 ^e,f^	769 ± 12 ^c^	12.5 ± 0.5 ^h,i,j^	5.84 ± 0.04 ^g,h^	23.70 ± 0.03 ^g^	76.30	-	4.33 ± 0.04 ^b,c,d^
NC140K15	18.1 ± 1.6 ^f,g,h,i^	-	19.2 ± 0.2 ^e^	6.35 ± 0.04 ^f,g^	26.10 ± 0.04 ^d^	73.90	-	3.89 ± 0.04 ^c,d,e^
NC140K20	13.3 ± 0.7 ^f,g,h,i^	-	10.7 ± 0.3 ^i,j^	9.76 ± 0.07 ^b^	19.40 ± 0.01 ^m^	80.60	-	4.71 ± 0.25 ^a,b^
NC140K25	6.13 ± 0.21 ^i^	697 ± 8 ^d^	10.2 ± 0.4 ^j^	11.62 ± 0.36 ^a^	19.90 ± 0.05 ^l^	80.10	-	5.25 ± 0.36 ^a^

nd = not detected. ^1^ The results are expressed as mean ± standard deviations. Lowercase letters in the same column represent significant differences (*p* < 0.05).

**Table 3:** Analysis results for Barlovento beans treated at 110 °C and 140 °C ^1^.

Code	Furosine (mg/100 g of Protein)	Acrylamide (µg/kg)	Furan (µg/kg)	Moisture (%)	L	Browning Index (100-L^•^)	pH	Ash (%)
NB0	225 ± 9 ^b^	nd ^j^	9.30 ± 0.20 ^f,g^	-	30.03 ± 0.00 ^a^	69.97	-	-
SB0	180 ± 8 ^c^	nd ^j^	9.83 ± 0.11 ^e,f^	-	-	-	-	-
NB110	189 ± 7 ^c^	154 ± 5 ^i^	9.16 ± 0.11 ^f,g^	-	-	-	-	-
SB110	41.9 ± 4.0 ^h,i^	1562 ± 14 ^a^	11.6 ± 0.3 ^d^	-	-	-	-	-
NB140	299 ± 5 ^a^	353 ± 11 ^e^	23.2 ± 0.5 ^a^	-	-	-	-	-
SB140	52.0 ± 3.9 ^g,h,i^	1113 ± 12 ^b^	16.0 ± 0.5 ^b^	-	-	-	-	-
NB110Na10	49.9 ± 0.4 ^g,h,i^	352 ± 7 ^e^	12.5 ± 0.4 ^c^	4.80 ± 0.49 ^f^	17.60 ± 0.03 ^o^	82.40	-	2.68 ± 0.04 ^g^
NB110Na15	98.3 ± 1.7 ^e^	-	10.6 ± 0.3 ^e^	5.14 ± 0.27 ^e,f^	22.20 ± 0.03 ^k^	77.80	-	4.16 ± 0.10 ^e,f^
NB110Na20	61.7 ± 5.7 ^f,g^	-	5.81 ± 0.08 ^j^	7.33 ± 0.12 ^c^	19.70 ± 0.02 ^n^	80.30	-	4.44 ± 0.19 ^c,d,e,f^
NB110Na25	36.3 ± 3.6 ^i^	307 ± 9 ^f^	6.22 ± 0.12 ^j^	4.85 ± 0.05 ^e,f^	25.90 ± 0.03 ^f^	74.10	-	4.30 ± 0.19 ^d,e,f^
NB110K10	3.49 ± 0.19 ^j^	370 ± 11 ^e^	7.91 ± 0.20 ^i^	7.08 ± 0.29 ^c^	26.30 ± 0.02 ^e^	73.70	-	3.82 ± 0.31 ^f^
NB110K15	115 ± 2 ^e^	-	8.28 ± 0.23 ^h,i^	6.34 ± 0.19 ^c,d^	25.20 ± 0.09 ^g^	74.80	-	4.25 ± 0.36 ^d,e,f^
NB110K20	55.2 ± 2.1 ^f,g,h^	-	4.86 ± 0.23 ^k^	9.12 ± 0.09 ^a,b^	20.00 ± 0.01^m^	80.00	-	4.81 ± 0.41 ^b,c,d,e^
NB110K25	2.03 ± 0.14 ^j^	134 ± 8 ^i^	1.77 ± 0 ^m^	7.05 ± 0.04 ^c^	19.50 ± 0.02 ^ñ^	80.50	-	4.82 ± 0.38 ^b,c,d,e^
NB140Na10	142 ± 9 ^d^	212 ± 4 ^h^	11.8 ± 0.1 ^c,d^	4.71 ± 0.34 ^f^	27.20 ± 0.03 ^c^	72.80	-	4.27 ± 0.05 ^d,e,f^
NB140Na15	61.2 ± 5.6 ^f,g^	-	7.91 ± 0.12 ^i^	5.08 ± 0.75 ^e,f^	22.50 ± 0.02 ^j^	77.50	-	4.01 ± 0.35 ^e,f^
NB140Na20	112 ± 1 ^e^	-	8.90 ± 0.28 ^g,h^	5.64 ± 0.07 ^d,e,f^	20.90 ± 0.04 ^l^	79.10	-	5.19 ± 0.17 ^a,b,c,d^
NB140Na25	139 ± 9 ^d^	250 ± 10 ^g^	11.7 ± 0.1 ^d^	5.24 ± 0.03 ^e,f^	20.90 ± 0.02 ^l^	79.10	-	5.28 ± 0.07 ^a,b,c^
NB140K10	116 ± 6 ^e^	527 ± 11 ^c^	10.6 ± 0.2 ^e^	5.82 ± 0.04 ^d,e^	27.40 ± 0.20 ^b^	72.60	6.6	4.51 ± 0.04 ^c,d,e,f^
NB140K15	147 ± 15 ^d^	-	4.94 ± 0.12 ^k^	7.10 ± 0.70 ^c^	26.60 ± 0.04 ^d^	73.40	7.2	4.52 ± 0.70 ^c,d,e,f^
NB140K20	71.4 ± 2.7 ^f^	-	3.32 ± 0.11 ^l^	8.63 ± 0.30 ^b^	24.90 ± 0.02 ^h^	75.10	8.1	5.90 ± 0.40 ^a^
NB140K25	72.8 ± 0.6 ^f^	446 ± 11 ^d^	3.40 ± 0.04 ^l^	9.96 ± 0.30 ^a^	23.40 ± 0.02 ^i^	76.60	8.7	5.48 ± 0.40 ^a,b^

nd = not detected. ^1^ The results are expressed as mean ± standard deviations. Lowercase letters in the same column represent significant differences (*p* < 0.05).

**Table 4:** Furosine, acrylamide and furan contents of Barlovento beans treated at 160 °C and 180 °C ^1^.

Code	Furosine (mg/100 g of Protein)	Acrylamide (µg/kg)	Furan (µg/kg)
NB16040	4.39 ± 0.43 ^c^	146 ± 5 ^h^	73.8 ± 3.5 ^g^
SB16040	-	398 ± 12 ^d^	329 ± 8 ^c^
NB16060	3.24 ± 0.17 ^c^	532 ± 11 ^b^	124 ± 4 ^d^
SB16060	-	263 ± 11 ^f^	680 ± 32 ^a^
NB18015	164 ± 9 ^a^	564 ± 11 ^a^	39.1 ± 1.4 ^i^
SB18015	-	97.0 ± 4.9 ^i^	81.7 ± 1.8 ^f^
NB18030	8.97 ± 0.81 ^c^	492 ± 10 ^c^	98.6 ± 5.2 ^e^
SB18030	-	358 ± 11 e	378 ± 10 ^b^
NB16040K20	-	178 ± 6 ^g^	10.8 ± 0.3 ^k^
NB16060K20	-	561 ± 11 ^a^	48.1 ± 0.8 ^h^
NB18015K20	39.0 ± 1.8 ^b^	140 ± 11 ^h^	13.2 ± 0.3 ^k^
NB18030K20	-	241 ± 7 ^f^	22.2 ± 1.0 ^j^

^1^ The results are expressed as mean ± standard deviations. Lowercase letters in the same column represent significant differences (*p* < 0.05).

## Data Availability

The original contributions presented in the study are included in the article, further inquiries can be directed to the corresponding author.
